# Supplementary figures and images for: Single Cell Transcriptomic Analyses Reveal the Impact of bHLH Factors on Human Retinal Organoid Development
Source: Front Cell Dev Biol. 2021 May 13;9:653305. doi: 10.3389/fcell.2021.653305 (PMC8155690; doi:10.3389/fcell.2021.653305)

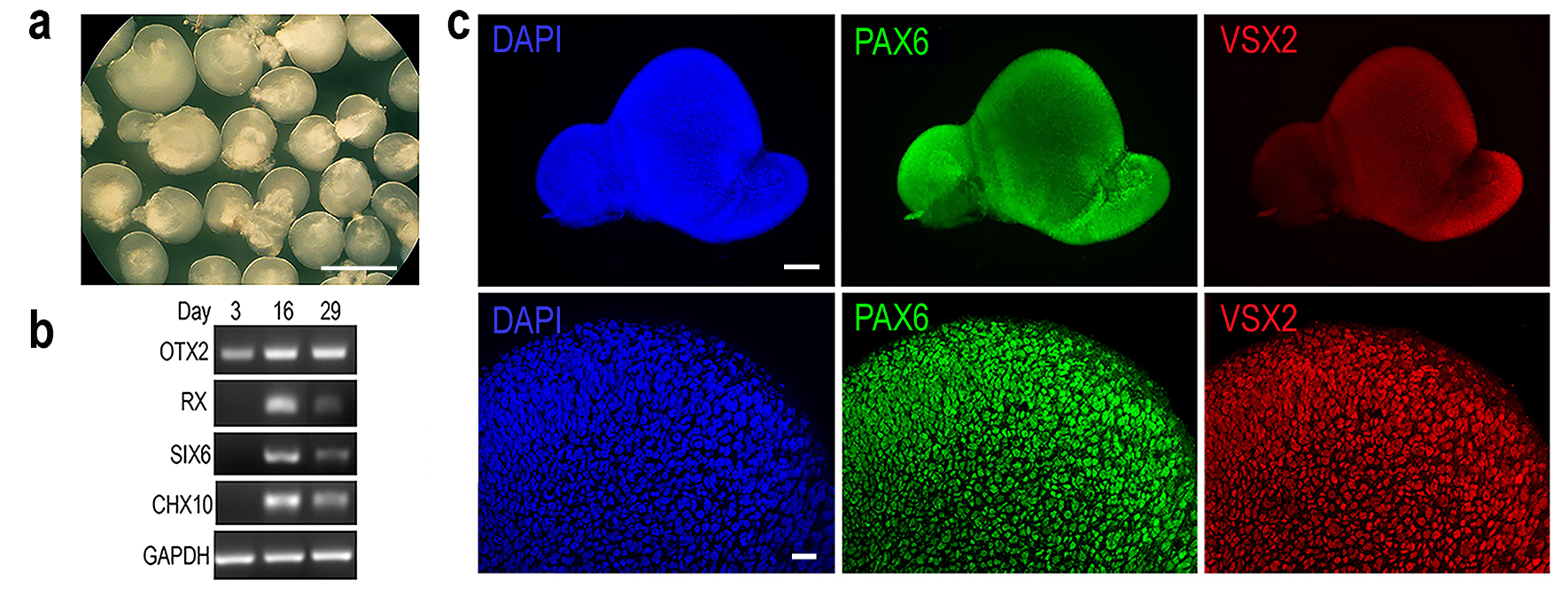

Supplement: Supplementary Figure 1 — Characterization of human ES cell-derived 3D retinal organoids. (a) Bright field image shows morphology of a group of H9 ES cell-derived 3D retinal organoid at Day 33.Scale bar, 1 mm. (b) RT-PCR assay detects expression of eye field and neural retina genes at Day 16 andDay 29 in H9 ES cell-derived cultures. (c) Whole mount images show a 3D retinal organoid coimmunolabeledfor PAX6 and VSX2 at Day 24. The top panels show low magnification images of theentire retinal organoid (scale bar, 100 μm), and the bottom panels show confocal images with nuclearlabeling of PAX6 and VSX2 in retinal progenitor cells (scale bar, 20 μm). [file Image_1.TIF]

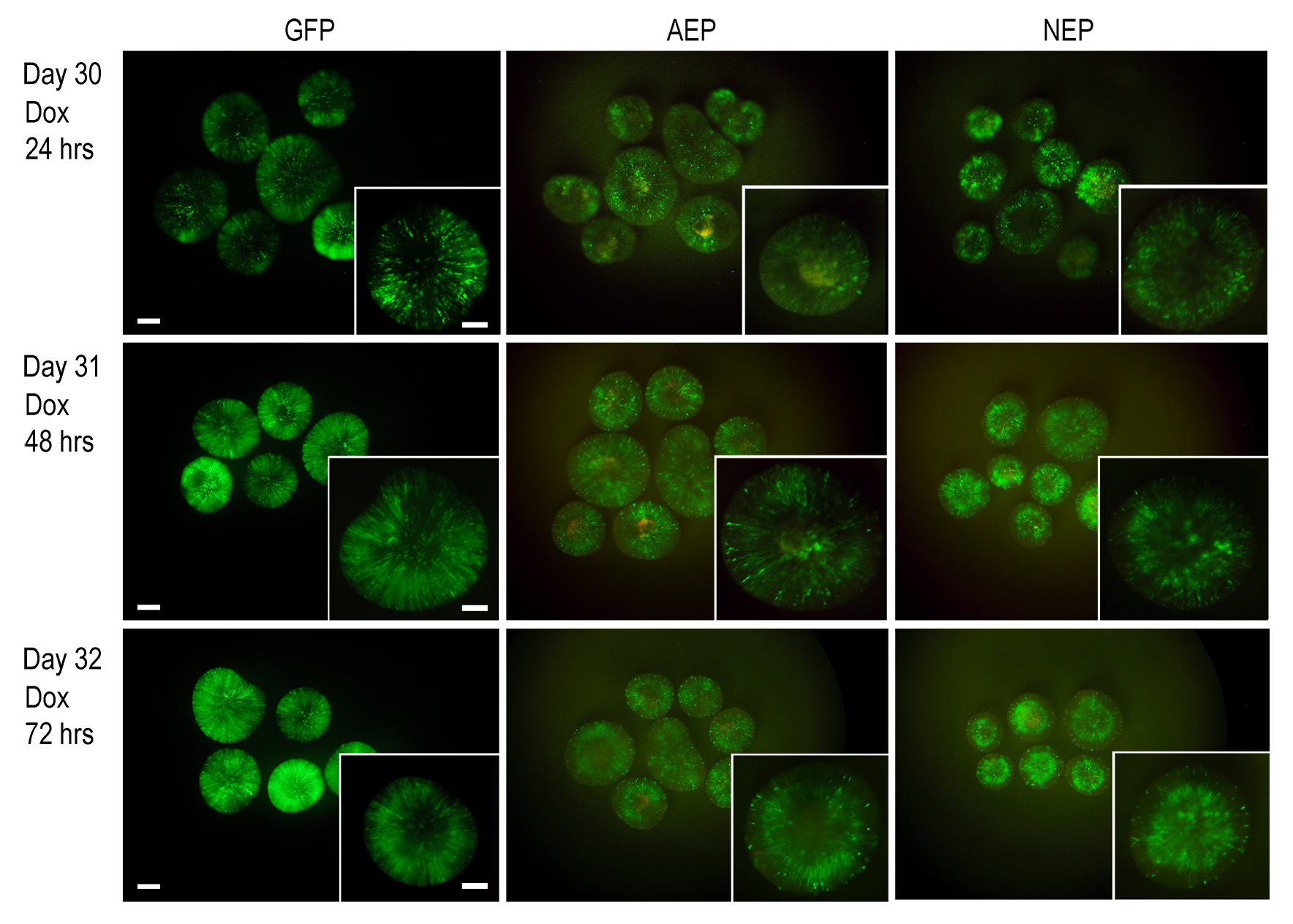

Supplement: Supplementary Figure 2 — Effect of Dox induction time course on retinal organoid development. Whole mount images show effects of different Dox induction durations on retinal organoiddevelopment. After 24-hour of Dox induction, both LV-AEP and LV-NEP infected cells showed atendency toward localizing to the inner layer, compared to the control LV-GFP virus infected retinalorganoids. This trend became more pronounced after 48-hour Dox induction. By 72-hour after theonset of Dox treatments, the majority of GFP+ cells were concentrated in the inner layer of the retinalorganoids. In contrast, most LV-GFP infected cells remained a ventricular zone distribution pattern after48- and 72-hour induction. Scale bars, 200 μm for the lower magnification; 100 μm for the insets. [file Image_2.TIF]

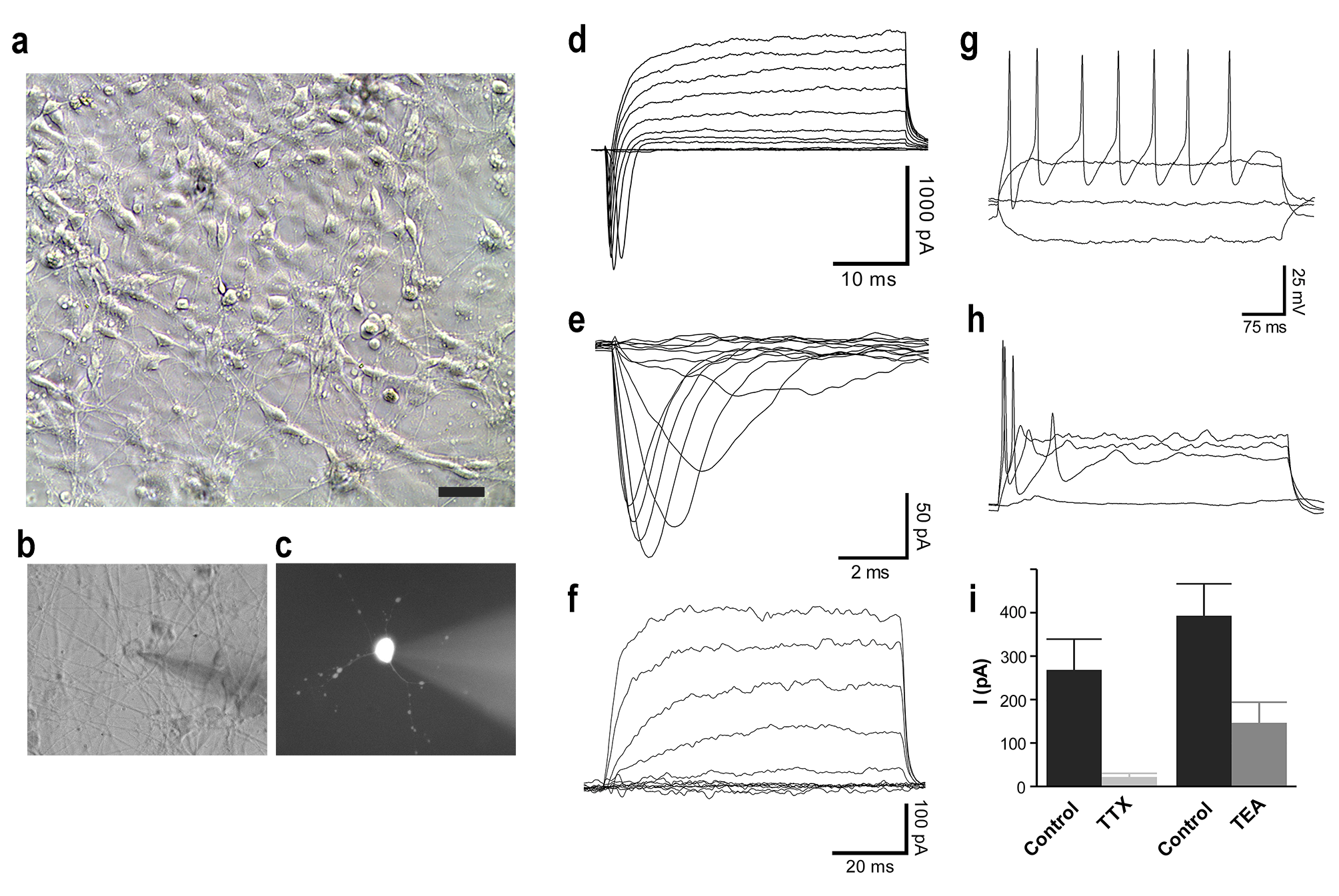

Supplement: Supplementary Figure 3 — Electrophysiological properties and functionality of Human H9 ES cell-derivedretinal neurons. (a) Bright field view of the dissociated cell culture derived from retinal organoids at Day 40. Scale bar, 20μm. (b, c) A neuron being patch recorded is shown in bright field (b) and after filling with lucifer yellowdye (c). (d-i) Whole cell patch clamp recording of dissociated neurons (Day 37-40) derived from H9 ESretinal organoids cultured as a monolayer. d Whole cell voltage clamp of a cell with multipolar neuritesstepped from −60 mV to +30 mV in 10 mV steps of 40 ms duration. (e) Example of well-clamped INaisolated by digital substraction following block with 100 nM TTX. Steps from −90 to +20 mV are shown. (f)Outward K+ currents isolated by digital substraction following 10 mM TEA application. Steps from −70 to+30 mV are shown. (g) Train of action potentials elicited with depolarizing current in current clampedcells having large INa. (h) Phasic action potential generation in cells having smaller INa. (i) Summary ofblock of peak INa and IK at +40 mV by TTX and TEA, respectively. TTX blocked 92% of the transient inwardcurrent (n=7) and TEA blocked 62% of the sustained outward current at +40 mV (n = 6). [file Image_3.TIF]

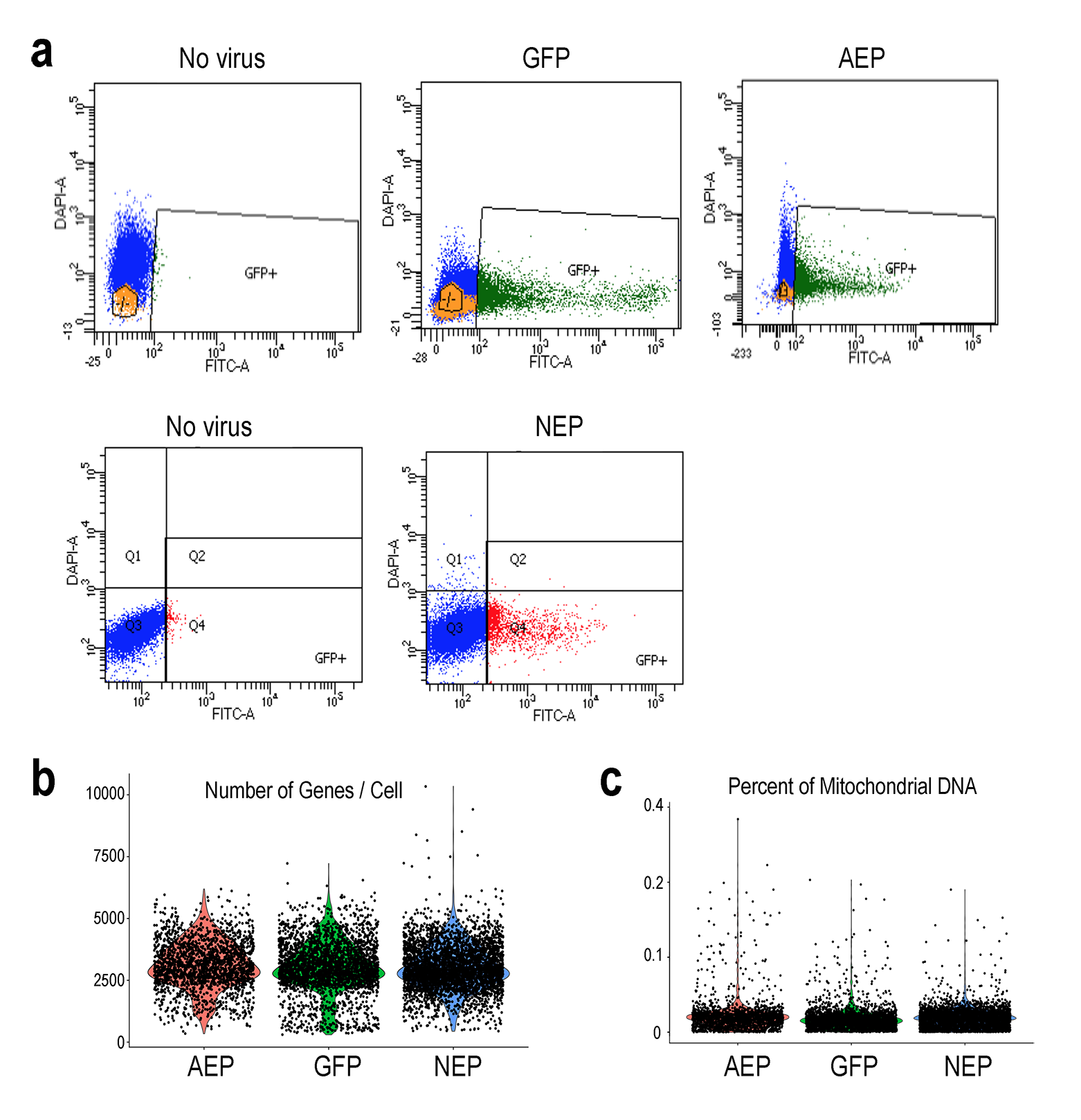

Supplement: Supplementary Figure 4 — Fluorescent activated cell sorting of lentivirus infected retinal organoids for single cell RNA-sequencing. (a) FACS profiles of dissociated retinal organoids infected with LV-GFP, LV-AEP, and LV-NEP between Day45-48 after 8 day ox treatment. Cells from non-infected retinal organoids were used as negativecontrols to set the thresholds for GFP+ cells. FACS enriched GFP+ cells were used for single cell RNA-seqanalyses. (b) Violin plots show the numbers of genes detected in single cell RNA-seq analyses using 10XGenomics Chromium and NovaSeq work flow. The cutoff used in this study was 2500 UMI per cell,resulting in mean gene per cell ranging from 2935-3079. For downstream analysis 3004 cells for LVGFP,2063 cells for LV-AEP, and 3909 cells for LV-NEP were used. (c) Violin plots show percentage ofmitochondrial encoded genes detected in single cell RNA-seq analyses using 10X Genomics Chromiumand NovaSeq work flow. The low rates (<0.03%) of transcripts from the mitochondrial genome indicatethat the transcripts analyzed in this study are from the nuclear genome. [file Image_4.TIF]

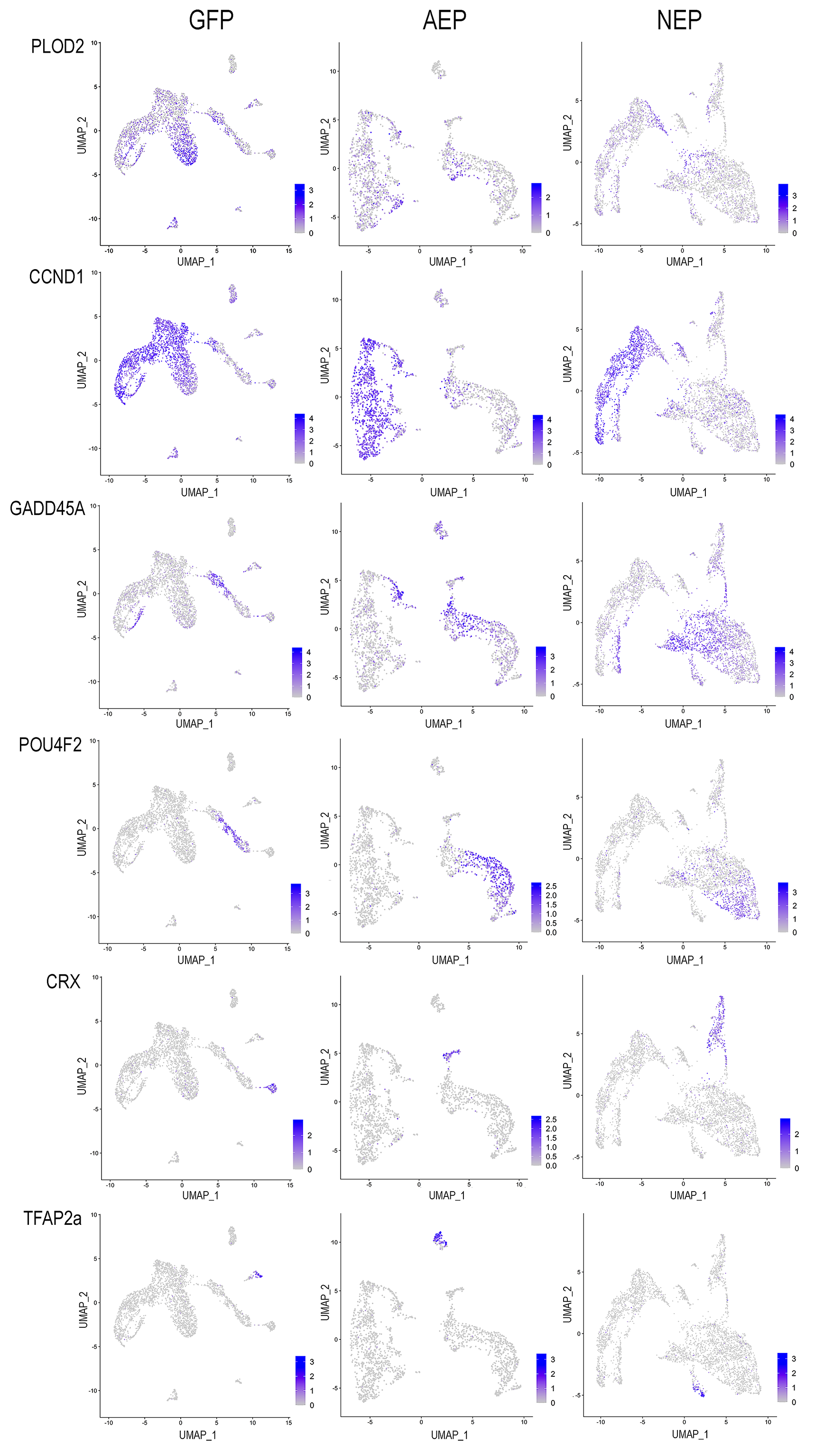

Supplement: Supplementary Figure 5 — Expression of known genes used to assign cell categories of cell clusters. Feature plots of known genes in LV-GFP, LV-AEP, or LV-NEP infected retinal organoids shown as UMAPs.Genes representing different cell category or states are used to assign cell cluster identities in Figure 5. [file Image_5.TIF]

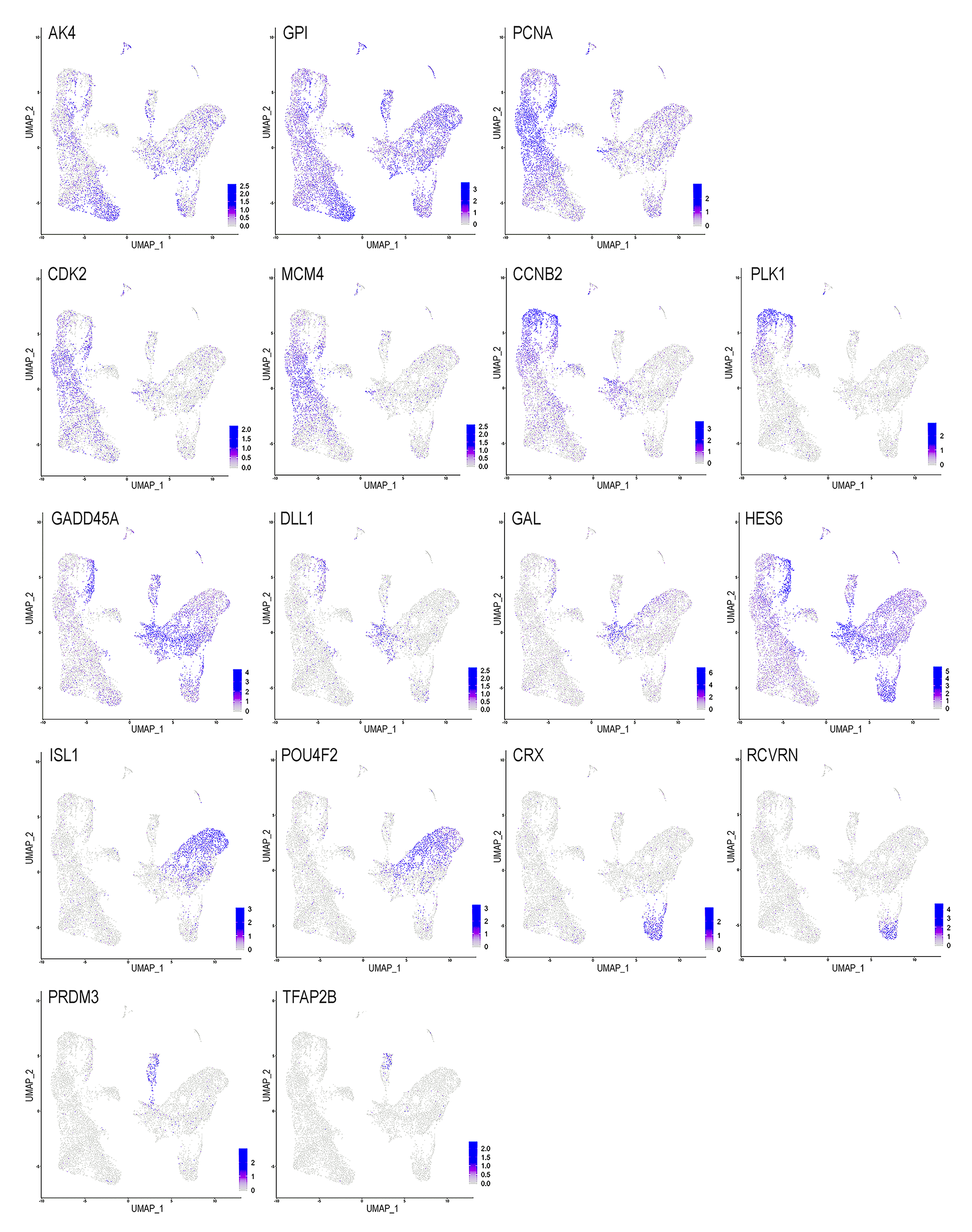

Supplement: Supplementary Figure 6 — Expression of featured known genes in combined sample clusters. Feature plots of known genes in the combined LV-GFP, LV-AEP, LV-NEP sample clusters shown asUMAPs. Genes representing different cell cycle phases and cell type categories are used to assigncluster identities in Figure 8. [file Image_6.TIF]

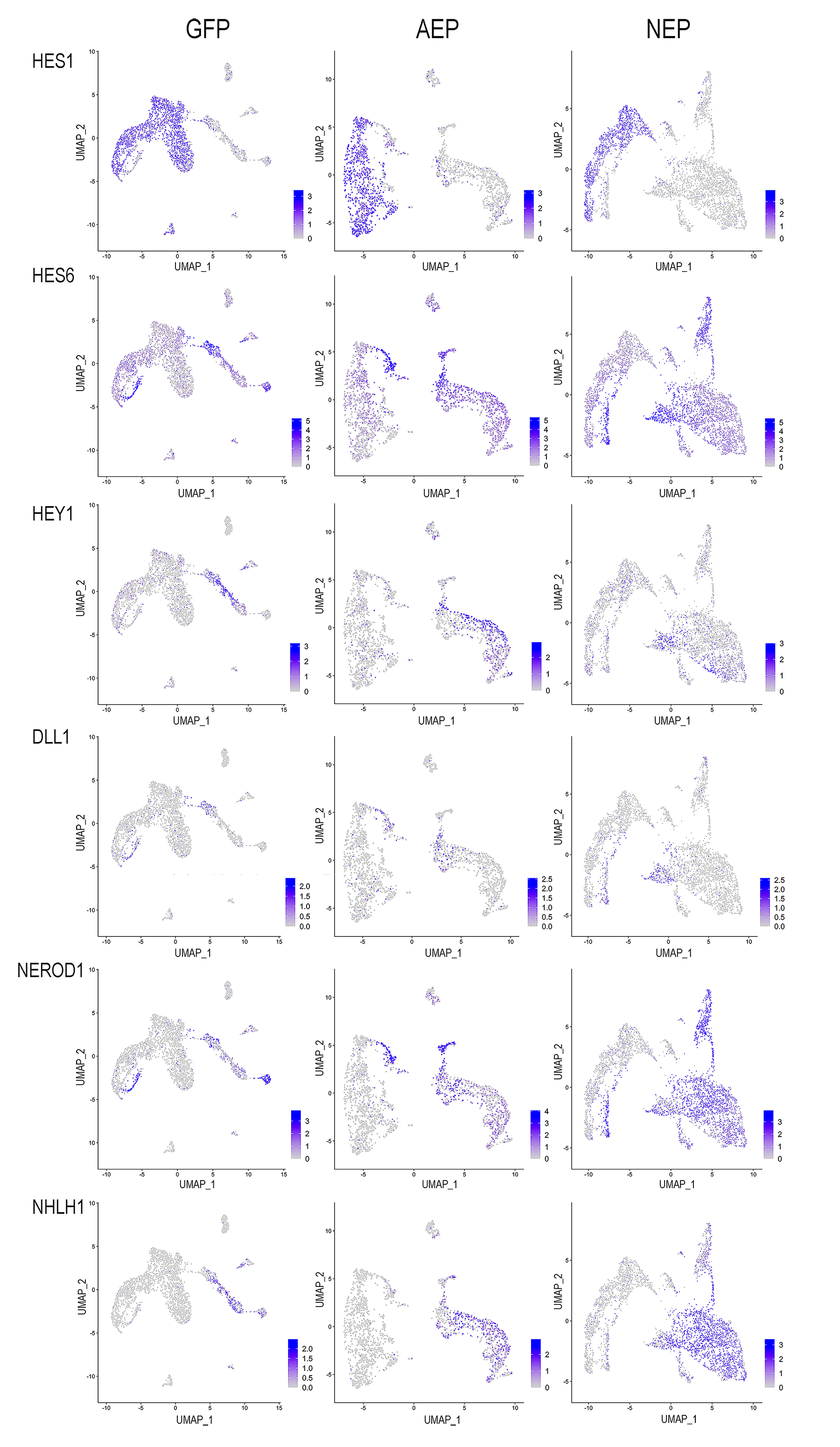

Supplement: Supplementary Figure 7 — Expression patterns of Notch signaling components and selected bHLH genes inlentiviral infected retinal organoid cells. Feature plots of selected genes quantified in Figure 9 are shown in UMAPs in LV-GFP, LV-AEP, and LVNEPinfected retinal organoid cells. HES1 Is predominantly expressed among neural stem cells andprogenitors. HES6, DLL1, and NEUROD1 are upregulated in exiting progenitors and neuroblasts. NHLH1is expressed among postmitotic cells. [file Image_7.TIF]

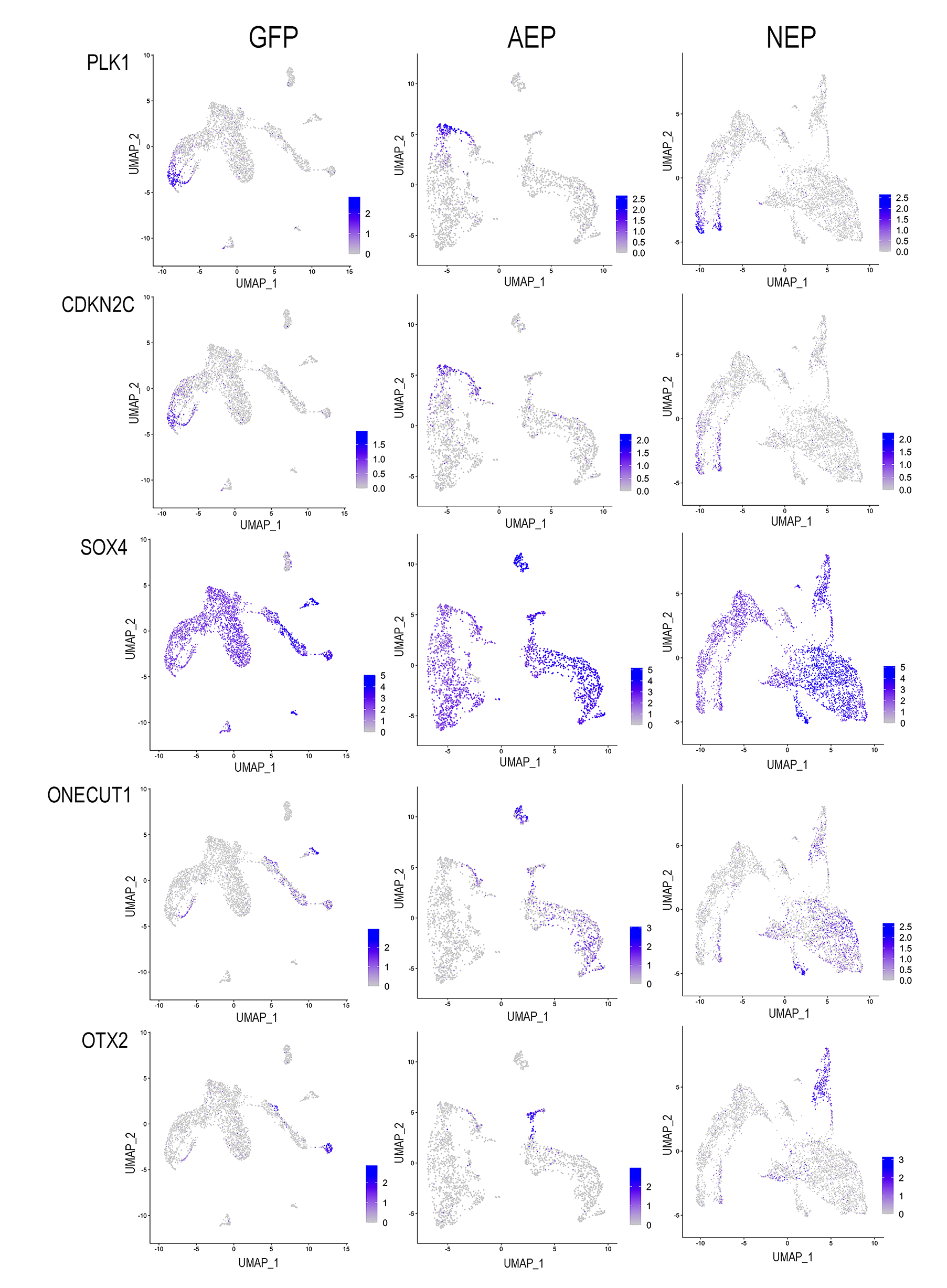

Supplement: Supplementary Figure 8 — Expression patterns of genes involved in cell cycle exit and early retinogenesis. Feature plots of selected genes involved in cell cycle exit (PLK1, CDKN2C) and differentiation of earlyretinal neurons (SOX4, ONECUT1, OTX2) are shown in UMAPs in LV-GFP, LV-AEP, and LV-NEP infectedretinal organoid cells. [file Image_8.TIF]
